# Serum SERPINB3/4 in Moderate‐to‐Severe Prurigo Nodularis: A Potential Biomarker for Disease Severity and Eosinophil‐Related Parameters

**DOI:** 10.1002/iid3.70263

**Published:** 2025-09-25

**Authors:** Jiali Wu, Kaoyuan Zhang, Yan Liao, Chener Yang, Jingyao Ge, Chenchen Wu, Bo Yu, Weilong Zhong, Yong Shao, Xia Dou

**Affiliations:** ^1^ Department of Dermatology Peking University Shenzhen Hospital, Institute of Dermatology, Shenzhen Peking University—The Hong Kong University of Science and Technology Medical Center Shenzhen China; ^2^ Shenzhen Key Laboratory for Translational Medicine of Dermatology, Biomedical Research Institute Shenzhen Peking University—The Hong Kong University of Science and Technology Medical Center, Guangdong Province Shenzhen China

**Keywords:** ELR, eosinophil, IGA, prurigo nodularis, SERPINB3/4

## Abstract

**Background:**

The available evidence increasingly indicates that SERPINB3/4 plays a pivotal role in the progression of inflammatory diseases and may serve as a valuable biomarker for atopic dermatitis and psoriasis. However, the expression of SERPINB3/4 in the serum of patients with moderate‐to‐severe prurigo nodularis (PN) remains poorly understood.

**Objective:**

To find a promising biomarker for monitoring the severity and activity of moderate‐to‐severe PN patients.

**Methods:**

A total of 41 patients with moderate‐to‐severe PN and 20 healthy subjects were included in the study. Enzyme­linked immunosorbent assay (ELISA) was performed to determine serum SERPINB3/4 expression. Cutaneous SERPINB3/4 expression was evaluated by immunohistochemistry (IHC). Serum SERPINB3/4 levels were correlated with PN disease severity and activity scores, hematological parameters, and systemic inflammatory markers.

**Results:**

SERPINB3/4 expression was found to be significantly increased in the serum and lesions of PN in comparison to healthy subjects. In patients with PN, the expression of serum SERPINB3/4 was significantly elevated in subjects with higher investigator global assessment (IGA) scores, while exhibiting a notable decline in patients with higher pruritus numeric rating scale (P‐NRS) scores. Positive correlations were observed between serum SERPINB3/4 levels and peripheral eosinophil count, eosinophil ratio, and eosinophil‐to‐lymphocyte ratio (ELR) in patients with PN.

**Conclusion:**

SERPINB3/4 expression was increased in PN sera and lesions. Serum SERPINB3/4 expression was positively correlated with PN severity and peripheral eosinophil‐related parameters, suggesting its potential as a promising biomarker for PN.

## 1 Introduction

Prurigo nodularis (PN) is a chronic inflammatory skin disorder characterized by intensely pruritic and hyperkeratotic nodules symmetrically distributed on the trunk and extensor surfaces of the extremities [1], which disproportionately affects African American patients [2]. PN has the highest pruritus intensity and attack frequency in common chronic pruritus skin diseases [3]. Immune and inflammatory dysregulation, neural dysregulation and fibrotic responses are involved in the development of PN. Dysregulated immune and inflammatory responses are instrumental in the pathogenesis of PN. The dermis in PN lesions is heavily populated by immune cells, such as T lymphocytes, eosinophils, and mast cells. These immune cells trigger inflammatory reactions and promote the abnormal proliferation of keratinocytes (KC) by secreting a range of itch‐inducing mediators and pro‐inflammatory cytokines, including IL‐4, IL‐5, IL‐13, and IL‐31. Additionally, neuropeptides like substance P, released by dermal neurons, intensify the inflammatory response and stimulate the excessive proliferation of KC [4]. The synergistic interactions of these processes collectively contribute to the intense itching and chronic inflammation of PN.

Recent cutaneous transcriptomic studies in PN have revealed Th22 and Th17 immune gene signatures shared among PN, psoriasis, and atopic dermatitis (AD), including SERPINB4 [5]. The members of the family of the serine‐protease inhibitors (SERPINS), B3(SERPINB3) and B4(SERPINB4), are also respectively known as squamous cell carcinoma antigen‐1(SCCA1) and SCCA2 and were originally discovered as tumor‐specific antigens [6]. SERPINB3 and SERPINB4 are highly homologous, sharing 91% of their amino acids [7]. Previous studies have identified SERPINB3/4 as tumor markers for certain squamous cell carcinomas [8], with potential utility as prognostic biomarkers in various cancers [9, 10].

Emerging evidence indicated upregulated SERPINB3/4 expression in serum and KCs of inflammatory skin diseases, including psoriasis and AD, where they regulate inflammatory responses through multiple pathways [11, 12, 13, 14]. Therefore, serum SERPINB4 level in psoriasis and AD is positively correlated with the clinical severity of patients and emerged as a useful biomarker indicating the severity of these patients [15]. Recent data has indicated that *SERPINB3* and *SERPINB4* were upregulated significantly in PN lesions compared with the nonlesional PN skin [16]. However, little is known about the relationship between serum SERPINB3/4 expression and PN severity. This study aims to elucidate the correlation between serum SERPINB3/4 levels and various disease activity and severity assessment indicators in moderate‐to‐severe PN patients.

## 2 Materials and Methods

### 2.1 Participants

All patients with PN were recruited from the Common Inflammatory Skin Diseases Cohort Study (ChiCTR2200058675) at Peking University Shenzhen Hospital between July 2020 and July 2023. The diagnosis of PN was primarily based on three core clinical criteria, as assessed by two independent dermatologists: (1) the presence of firm, nodular lesions; (2) pruritus lasting at least 6 weeks; and (3) history and/or signs of repeated scratching, picking, or rubbing [17]. A skin biopsy is generally reserved for cases where treatment outcomes are unsatisfactory. Healthy skin samples were obtained from lipoma patients undergoing outpatient surgery in the Department of Dermatology at Peking University Shenzhen Hospital. The samples were collected from normal skin adjacent to lipomas located on the extensor sides of the limbs. Control samples were selected to match the gender and age of the PN patients. All participants were Han Chinese and aged 18 years or older. The study was approved by the Ethical Committee of Peking University Shenzhen Hospital (No. 2022‐022) and was conducted according to the Declaration of Helsinki principles. Written informed consent was obtained from all participants.

### 2.2 Clinical Assessment

Patient clinical data were collected, including demographic data, serum total IgE levels, complete blood count data, investigator's global assessment scores (IGA; range 0–4, 0 = clear, 1 = almost clear, 2 = mild, 3 = moderate, and 4 = severe) [18], prurigo activity score (PAS; range 0–4, 0 = 0%, 1 = 1%–25%, 2 = 26%–50%, 3 = 51%–75%, and 4 = 76%–100%) [19], the maximum pruritus NRS score (P‐NRSmax; representing the worst itch intensity) in the past week, moderate pruritus (3 < P‐NRSmax score ≤ 6), severe pruritus (6 < P‐NRSmax score ≤ 10) [20], dermatology life quality index (DLQI), Hospital Anxiety and Depression Scale (HADS), and Pittsburgh Sleep Quality Index (PSQI).

Eosinophil‐, neutrophil‐, monocyte‐, platelet‐ and basophil‐to‐lymphocyte ratios (ELR, NLR, MLR, PLR and BLR), systemic immune‐inflammation index (SII) and pan‐immune‐inflammation value (PIV) were calculated as follows: ELR = eosinophil count/lymphocyte count; NLR = neutrophil count/lymphocyte count; MLR = monocyte count/lymphocyte count; PLR = platelet count/lymphocyte count; BLR = basophil count/lymphocyte count; SII = (neutrophil count × platelet count)/lymphocyte count; PIV = (neutrophil count × platelet count × monocyte count)/lymphocyte count [21, 22, 23].

### 2.3 Enzyme­Linked Immunosorbent Assay (ELISA)

Serum specimens were collected and stored at −80°C. Concentrations of SERPINB3/4 in serum were measured using a commercially available ELISA with standard kits according to the manufacturer's instructions (ELISA Kit for Squamous Cell Carcinoma Antigen 1/2 (SCCA1/SCCA2), Cloud‐Clone Corp., China).

### 2.4 Immunohistochemistry (IHC)

Paraffin‐embedded tissue sections (5 μm) from cutaneous biopsies were dewaxed and dehydrated with xylene and graded ethanol. Microwave the slides in a boiling citric acid buffer (PH 6.0) over low heat for 17 min. Slides were dripped with H_2_O_2_ to inactivate endogenous peroxidase and then were blocked by the blocking solution composed of 4% goat serum and 4% BSA for 1 h. The slides were incubated with the anti‐SERPINB3/4 antibody (ab254255, 1:500) at 4°C overnight, and the secondary antibody labeled with horseradish peroxidase (HRP)‐labeled goat anti‐rabbit IgG was incubated at room temperature for 1 h the next day. All slides were developed using a DAB kit (ZSGB‐BIO, China) and counterstained. Slides were then digitally scanned by Aperio CS2 scanner (Leica, Germany). These scanned images were then processed and analyzed using QuPath software, and representative images were exported for further quantitative analysis in ImageJ. The percentage of positively stained area was quantified in at least three randomly selected fields per section by the ImageJ software.

### 2.5 Statistical Analysis

Data are presented as means ± standard error of mean (SEM). Statistical significance between groups were determined using the *χ* ^2^ test for categorical data and using the Mann–Whitney *U* test for continuous data with SPSS 22.0. Correlations were analyzed by Pearson's correlation test for parametric data and Spearman's rho correlation test for non‐parametric data. *p* < 0.05 was considered statistically significant.

## 3 Results

### 3.1 Population Characteristics and Serum SERPINB3/4 Expression

**Figure 1:**
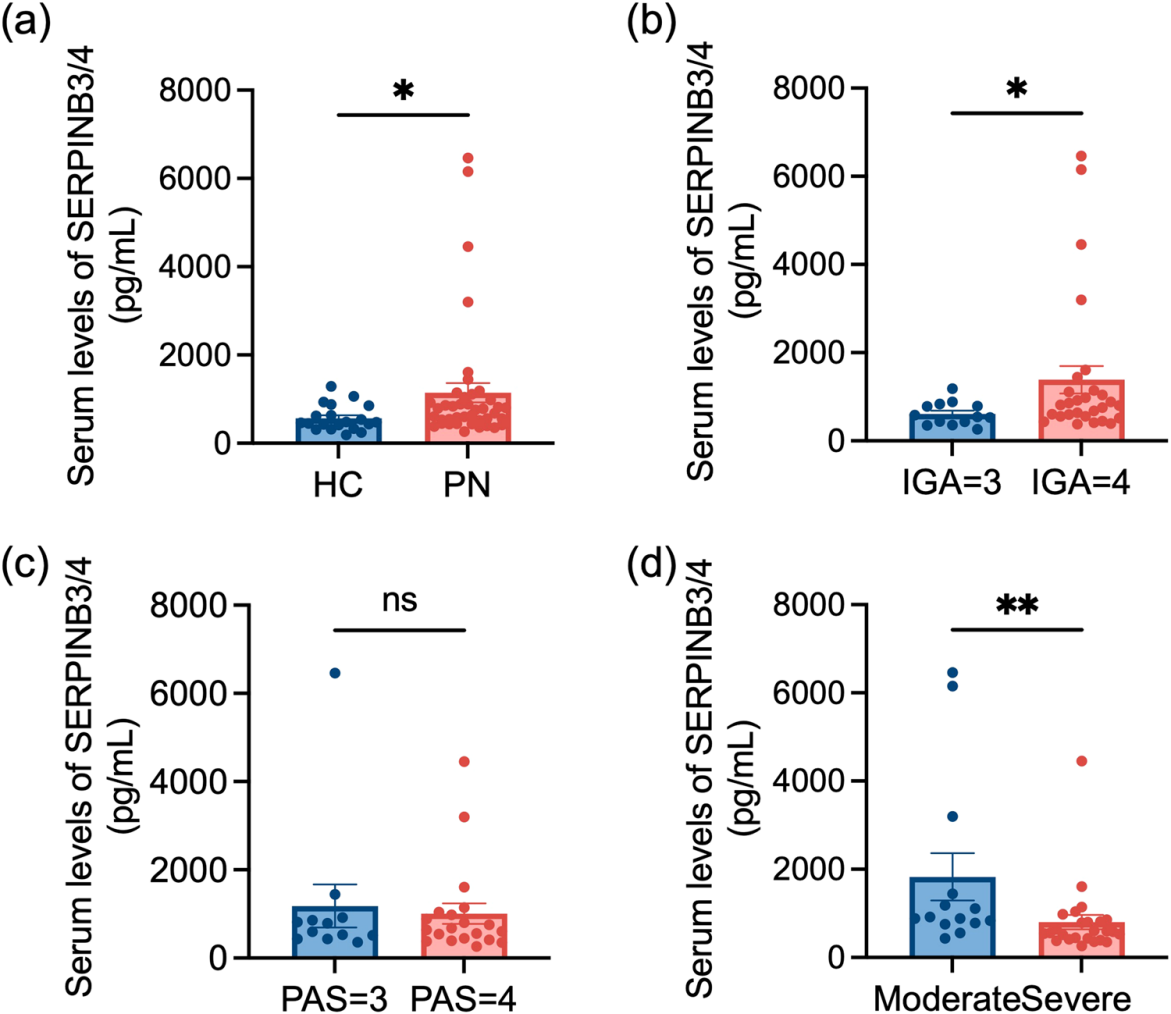
Serum SERPINB3/4 expression in moderate‐to‐severe PN patients. Serum SERPINB3/4 levels were measured using ELISA and compared across the following groups: (a) PN patients (*n* = 41) vs. healthy subjects (*n* = 20), (b) PN patients with an IGA score of 3 (*n* = 13) vs. those with an IGA score of 4 (*n* = 28), (c) PN patients with a PAS score of 3 (*n* = 12) vs. those with a PAS score of 4 (*n* = 20), and (d) PN patients with moderate pruritus (3 < P‐NRSmax score ≤ 6, *n* = 14) vs. those with severe pruritus (6 < P‐NRSmax score ≤ 10, *n* = 25). IGA, investigator's global assessment; PAS, prurigo activity and severity score; P‐NRS, pruritus numeric rating scale, P‐NRS max, the maximum P‐NRS score in the past week; PN, prurigo nodularis. Data are presented as means ± SEM. *p < 0.05, **p < 0.01, ns: no significance.

A total of 41 PN patients and 20 healthy subjects were enrolled in this study. Based on IGA scores [24], all PN patients had moderate‐to‐severe disease with severe pruritus and high disease activity. Table 1 summarizes the demographic and clinical characteristics of the participants. No significant differences were observed in age (*p* = 0.7675) or gender distribution (*p* = 0.5254) between the two groups. Serum SERPINB3/4 expression levels were significantly elevated (*p* = 0.0205) in the PN group compared to the healthy group (Figure 1a).

**Table 1:** The demographic and clinical characteristics of PN patients and healthy subjects.

Characteristics	PN patients	Healthy subjects	*p*
Age (years)	42.29 ± 2.84	40.90 ± 3.33	0.7675
Percentage of males	22 (53.7%)	9 (45%)	0.5254
Disease duration (years)	5.53 ± 1.00	/	
A history of atopy	20 (48.8%)	/	
Clinical scores			
IGA	3.68 ± 0.07	/	
PAS	3.22 ± 0.14	/	
P‐NRSmax	7.20 ± 0.31	/	
DLQI	11.56 ± 0.95	/	
PSQI	7.33 ± 0.52	/	
HADS	10.32 ± 0.90	/	

Abbreviations: DLQI, dermatology life quality index; HADS, hospital anxiety and depression scale; IGA, investigator's global assessment; PAS, prurigo activity and severity score; P‐NRS, pruritus numeric rating scale; P‐NRS max, the maximum P‐NRS score in the past week; PSQI, Pittsburgh sleep quality index.

### 3.2 Serum SERPINB3/4 Expression Was Increased in PN Patients With High IGA Score While Decreased in PN Patients With Severe Pruritus

To evaluate the potential of serum SERPINB3/4 as a biomarker for PN severity and activity, patients were stratified by IGA, PAS, and P‐NRSmax scores (over the past week), and differences in serum SERPINB3/4 expression were compared across subgroups.

In PN patients with an IGA score of 3 (IGA 3) and an IGA score of 4 (IGA 4), a significant increase (*p* = 0.0338) in serum SERPINB3/4 levels was observed in the IGA 4 group with a higher number of nodules (Figure 1b). As shown in Figure 1c, there was no significance but a decreasing tendency (*p* = 0.9847) in serum SERPINB3/4 expression between the group of PAS score of 3 (PAS 3) and the group of PAS score of 4 (PAS 4). Interestingly, serum SERPINB3/4 expression was significantly decreased (*p* = 0.0043) in PN patients with severe pruritus compared to PN patients with moderate pruritus (Figure 1d).

**Figure 2:**
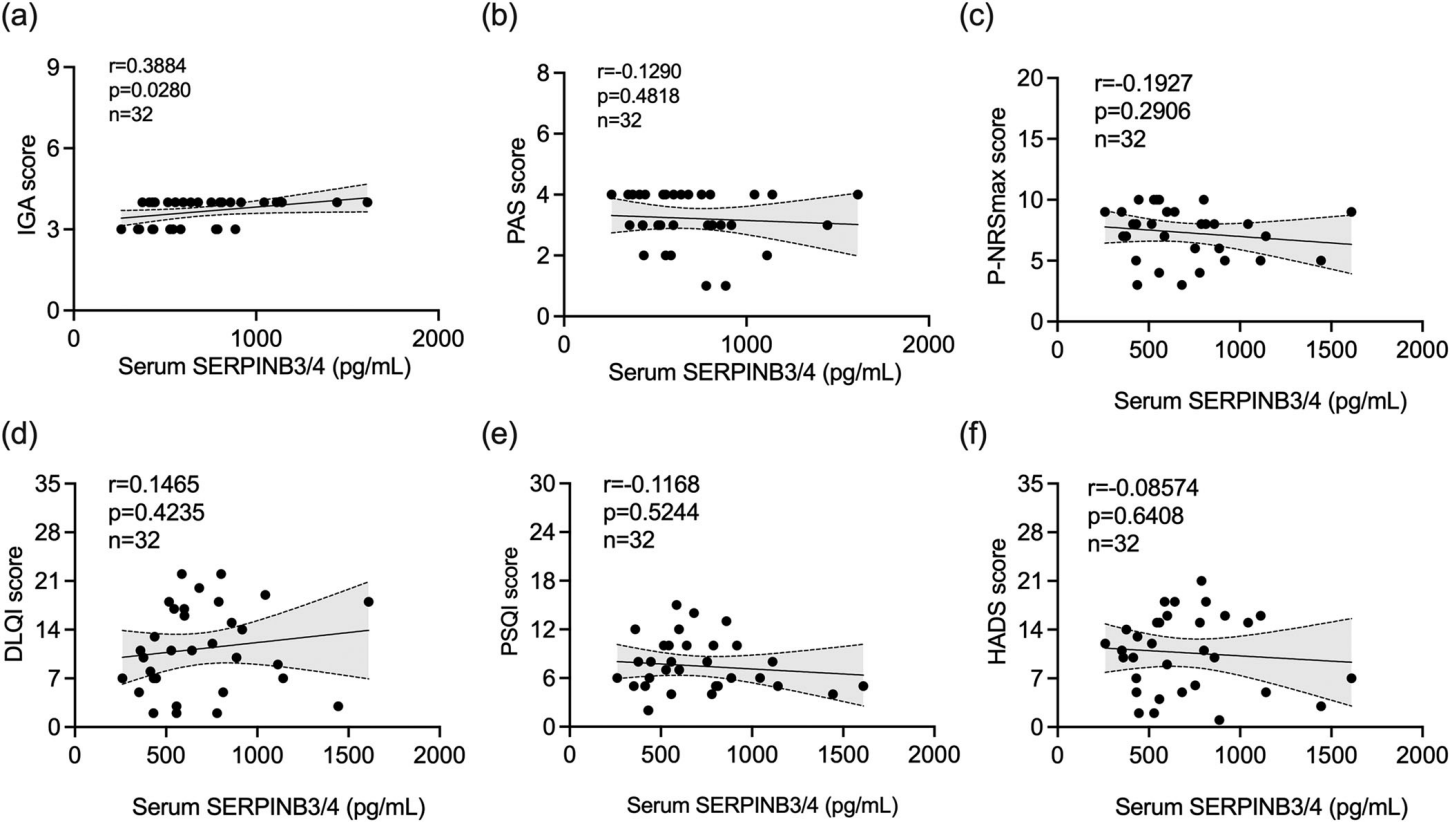
Correlation between serum SERPINB3/4 expression levels and clinical scores in PN. Correlation of SERPINB3/4 protein levels in the serum of PN patients with the IGA scores (a), PAS scores (b), maximum P‐NRS scores in the past week (c), DLQI scores (d), PSQI scores (e), and HADS scores (f). Correlations were analyzed by Pearson's correlation test for parametric data and Spearman's rho correlation test for non‐parametric data. DLQI, dermatology life quality index; HADS, hospital anxiety and depression scale; IGA, investigator's global assessment; PAS, prurigo activity and severity score; P‐NRS, pruritus numeric rating scale; PN, prurigo nodularis; PSQI, Pittsburgh sleep quality index.

A positive correlation (*r* = 0.3884, *p* = 0.0280) was observed between serum SERPINB3/4 levels and IGA scores (Figure 2a). However, no significant correlations were found between serum SERPINB3/4 levels and PAS scores (*r* = −0.1290, *p* = 0.4818), P‐NRS scores (*r* = −0.1927, *p* = 0.2906), DLQI scores (*r* = 0.1465, *p* = 0.4235), PSQI scores (*r* = −0.1168, *p* = 0.5244), or HADS scores (*r* = −0.08574, *p* = 0.6408) in PN patients (Figure 2b–f).

### 3.3 Hematological Parameters and Systemic Inflammatory Markers in PN Patients

Hematological parameters were compared across subgroups with varying PN severity and activity, as shown in Table 2. The results showed that eosinophil count (*p* = 0.0025), eosinophil ratio (*p* = 0.0098) and basophil ratio (*p* = 0.0457) were significantly increased in patients with IGA 4 compared to patients with IGA 3. In addition, monocyte count (*p* = 0.0239) and eosinophil count (*p* = 0.0240) were significantly elevated in PN patients with PAS 4 in comparison to the PAS 3 group.

**Table 2:** Descriptive hematological parameters of moderate‐to‐severe PN patients.

Variable	IGA score		*p*	PAS score		*p*	P‐NRSmax score		*p*
	3	4		3	4		Moderate	Severe	
tIgE (kU/L)	676.80 ± 306.10	643.00 ± 211.00	0.6689	432.50 ± 195.20	731.70 ± 292.60	0.535	947.00 ± 404.30	398.50 ± 117.10	0.259
WBC (×10^9^/L)	6.49 ± 0.45	6.78 ± 0.45	0.7417	6.05 ± 0.45	7.03 ± 0.49	0.186	6.95 ± 0.49	6.46 ± 0.37	0.429
N (×10^9^/L)	3.96 ± 0.30	4.18 ± 0.36	0.8568	3.74 ± 0.38	4.31 ± 0.39	0.335	4.36 ± 0.38	3.92 ± 0.28	0.353
L (×10^9^/L)	1.82 ± 0.20	1.63 ± 0.14	0.1813	1.59 ± 0.17	1.66 ± 0.19	0.752	1.69 ± 0.17	1.68 ± 0.15	0.745
M (×10^9^/L)	0.48 ± 0.05	0.49 ± 0.04	0.9812	0.43 ± 0.06	0.55 ± 0.04	0.024*	0.54 ± 0.06	0.46 ± 0.03	0.218
EOS (×10^9^/L)	0.19 ± 0.03	0.54 ± 0.13	0.0025**	0.24 ± 0.04	0.59 ± 0.16	0.024*	0.31 ± 0.06	0.46 ± 0.13	0.734
BASO (×10^9^/L)	0.04 ± 0.01	0.05 ± 0.00	0.2764	0.04 ± 0.01	0.05 ± 0.01	0.168	0.05 ± 0.01	0.04 ± 0.00	0.434
N (%)	61.39 ± 2.62	60.61 ± 2.02	0.8164	61.11 ± 2.84	60.92 ± 2.36	0.959	62.24 ± 2.37	60.41 ± 1.97	0.568
L (%)	27.69 ± 2.12	24.79 ± 1.69	0.3022	26.97 ± 2.43	23.66 ± 1.92	0.297	24.73 ± 2.22	25.98 ± 1.60	0.646
M (%)	7.37 ± 0.48	7.42 ± 0.42	0.8815	7.02 ± 0.64	7.98 ± 0.47	0.232	7.79 ± 0.63	7.31 ± 0.38	0.492
EOS (%)	3.02 ± 0.54	6.46 ± 1.30	0.0098**	4.28 ± 0.71	6.70 ± 1.53	0.164	4.53 ± 0.92	5.62 ± 1.23	0.482
BASO (%)	0.44 ± 0.08	0.73 ± 0.10	0.0457*	0.63 ± 0.08	0.75 ± 0.12	0.637	0.64 ± 0.09	0.67 ± 0.09	0.96
RBC (×10^12^/L)	4.77 ± 0.17	4.70 ± 0.11	0.7067	4.57 ± 0.12	4.73 ± 0.14	0.44	4.83 ± 0.15	4.66 ± 0.11	0.375
PLT (×10^9^/L)	237.00 ± 25.99	253.00 ± 10.31	0.5752	223.70 ± 13.12	260.20 ± 12.66	0.068	253.70 ± 21.45	246.90 ± 11.56	0.760
Hgb (g/L)	139.30 ± 4.49	137.80 ± 4.58	0.8358	136.30 ± 3.87	142.80 ± 4.63	0.341	139.90 ± 5.89	137.90 ± 3.66	0.286

Abbreviations: BASO, basophil; EOS, eosinophil; Hgb, hemoglobin; IGA, investigator's global assessment; L, lymphocyte; M, monocyte; N, neutrophil; PAS, prurigo activity and severity score; P‐NRS, pruritus numeric rating scale; P‐NRS max, the maximum P‐NRS score in the past week; tIgE, total IgE; PLT, platelet; RBC, red blood cell; WBC, white blood cell.

* *p* < 0.05

** *p* < 0.01.

**Figure 3:**
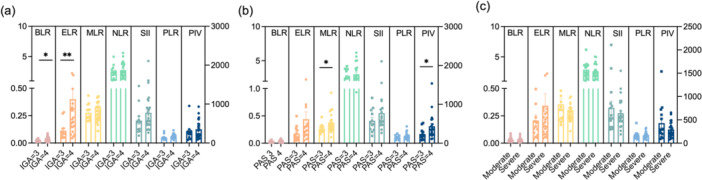
Systemic inflammatory markers in various moderate‐to‐severe PN patients. BLR, ELR, MLR, NLR, SII, PLR and PIV in PN patients respectively (a) with an IGA score of 3 (*n* = 13) and 4 (*n* = 24), (b) with a PAS score of 3 (*n* = 12) and 4 (*n* = 20) as well as (c) with moderate pruritus (3 < P‐NRSmax score ≤ 6, n = 14) and severe pruritus (6 < P‐NRSmax score ≤ 10, *n* = 25). The *Y* values of BLR, ELR, MLR and NLR were plotted on the left *Y* axis while *Y* values of SII, PLR and PIV were plotted on the right *Y* axis. BLR, basophil‐to‐lymphocyte ratio; ELR, eosinophil‐to‐lymphocyte ratio; MLR, monocyte‐to‐lymphocyte ratio; NLR, neutrophil‐to‐lymphocyte ratio; IGA, investigator's global assessment; PAS, prurigo activity and severity score; P‐NRS, pruritus numeric rating scale; P‐NRS max, the maximum P‐NRS score in the past week; SII, systemic immune‐inflammation index; PLR, platelet‐to‐lymphocyte ratio; PIV, pan‐immune‐inflammation value. Data are presented as means ± SEM. *p < 0.05, **p < 0.01.

Systemic inflammatory markers, including ELR, NLR, MLR, PLR, BLR, SII, and PIV, were compared across subgroups. The results demonstrated that ELR (*p* = 0.0017) and BLR (*p* = 0.0466) were significantly higher in the IGA 4 group than in the IGA 3 group (Figure 3a). MLR (*p* = 0.0324) and PIV (*p* = 0.0189) were significantly increased in the PAS 4 group of compared to the PAS 3 group (Figure 3b). Conversely, no differences in systemic inflammatory markers were observed between two groups stratified by P‐NRSmax scores in the past week (Figure 3c).

### 3.4 Serum SERPINB3/4 Positively Correlated With Eosinophils‐Associated Parameters

**Figure 4:**
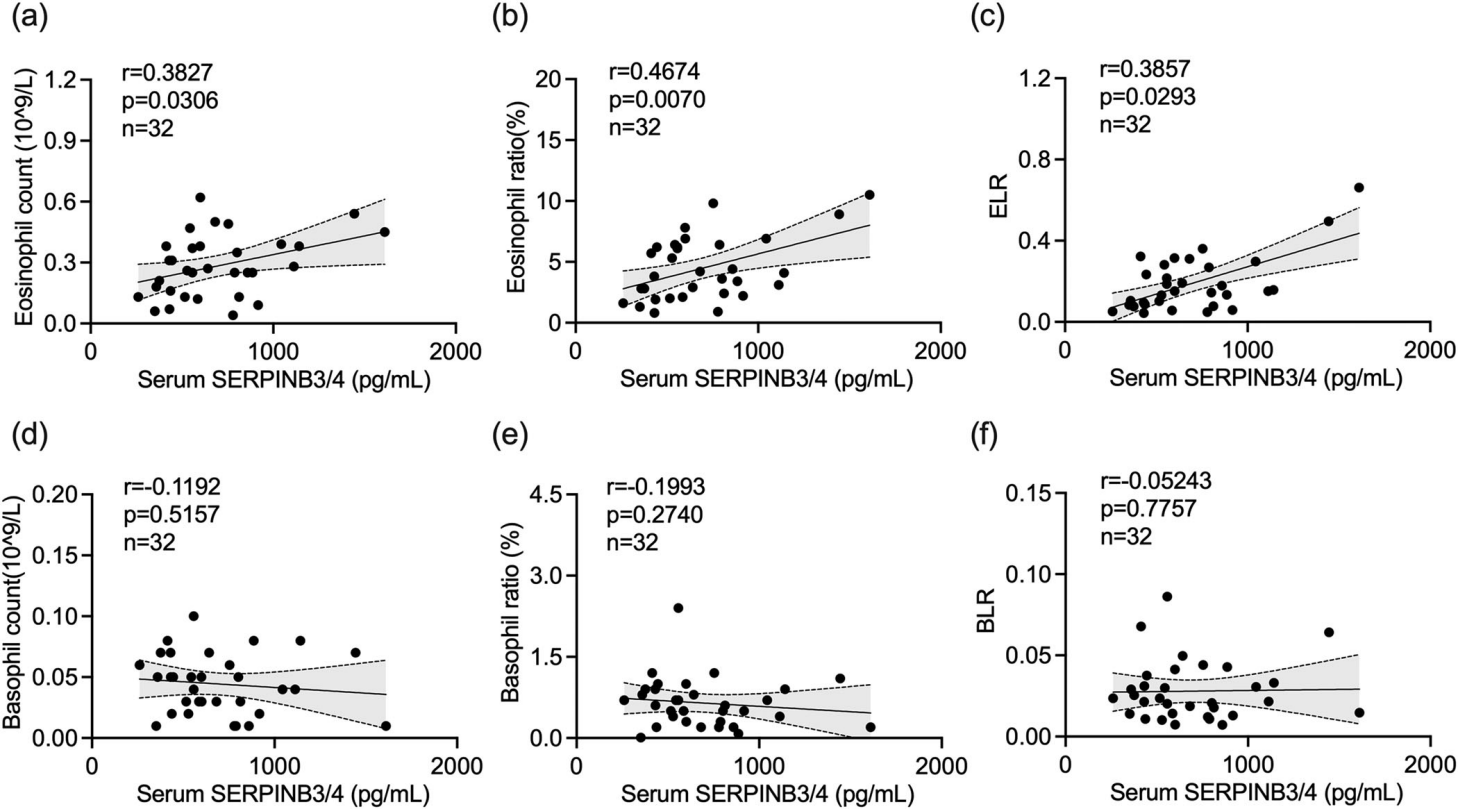
Correlation between serum SERPINB3/4 expression levels and inflammatory parameters in PN. The correlation analysis of serum SERPINB3/4 in all PN patients with eosinophil count (a), eosinophil percentage (b), ELR (c), basophil count (d), basophil percentage (e) and BLR (f). Correlations were analyzed by Pearson's correlation test for parametric data and Spearman's *ρ* correlation test for non‐parametric data. BLR, basophil‐to‐lymphocyte ratio; ELR, eosinophil‐to‐lymphocyte ratio; PN, prurigo nodularis.

As noted above, serum SERPINB3/4 levels and relevant markers of eosinophils and basophils were significantly elevated in PN patients with IGA 4. To explore the relationship between serum SERPINB3/4 expression and systemic inflammatory markers, correlation analyses were performed. The results showed that serum SERPINB3/4 expression was positively associated with eosinophil count (*r* = 0.3827, *p* = 0.0306), eosinophil percentage (*r* = 0.4674, *p* = 0.007) of eosinophils, and ELR (*r* = 0.3857, *p* = 0.0293) (Figure 4a–c). However, no significant correlations were observed among basophil count (*r* = −0.1192, *p* = 0.5157), basophil percentage (*r* = −0.1993, *p* = 0.274), and BLR (*r* = −0.05243, *p* = 0.7757) (Figure 4d–f).

### 3.5 Epidermal Expression of SERPINB3/4 Was Enhanced in PN

To determine whether SERPINB3/4 expression in PN nodules aligned with serum levels, we assessed SERPINB3/4 expression in PN lesions and healthy skin samples using IHC. In comparison to the healthy controls (HCs), SERPINB3/4 expression was markedly elevated (*p* = 0.0003) in PN, predominantly in the KCs of epidermis (Figure S1a,b).

## 4 Discussion

Our study is the first to reveal the expression of SERPINB3/4 in the serum of patients with moderate‐to‐severe PN and its correlation with type 2 inflammatory markers. In our study, the expression of SERPINB3/4 was significantly increased in the serum of PN patients with an IGA 4 while decreased in severe pruritus. In addition, serum levels of SERPINB3/4 in patients with moderate‐to‐severe PN were positively correlated with peripheral eosinophil counts and the inflammatory indicator ELR, suggesting that SERPINB3/4 may play a role in the inflammatory response underlying PN nodule formation.

Originally found to be highly expressed in multiple tumors, SERPINB3/4 was later found to be elevated in the serum of patients with AD and psoriasis as an inflammatory indicator and was thought to be associated with disease activity in AD and psoriasis [15, 25]. In our study, compared with HCs, serum SERPINB3/4 expression in PN patients with IGA 4 was significantly elevated, which was consistent with the increased expression of SERPINB4 in AD serum samples. Interestingly, among AD subtypes, serum SERPINB4 expression in the prurigo type was lower than that in the erythroderma type and widespread type [26]. However, in our study, no significant difference was observed between PN patients with and without an atopic history (Figure S2). We hypothesize that while PN shares some immune features with AD, such as Th2 responses, its more complex pathology involving Th17 and Th22 pathways may lead to SERPINB3/4 expression being regulated by cytokines like IL‐22, independent of atopic history.

The histopathological features of PN include abnormal proliferation of the epidermis, dermal fibrosis, and inflammatory cell infiltration [27]. Dysfunctional KCs in the PN epidermis were regulated by a variety of locally infiltrated immune cells and inflammatory cytokines. SERPINB3/4 expression was enhanced in the KCs of epidermis of PN, suggesting that SERPINB3/4 might be involved in the abnormal function of PN epidermis. SERPINB3/4 was also highly deposited in the KCs of epidermis of the AD and psoriatic lesion [15, 28]. SERPINB3/4 was a downstream molecule regulated by Th2, Th17, and Th22‐related inflammatory factors in the AD lesion and psoriatic lesion [15]. New evidence has shown that the immune signatures of both Th17 and Th22 were present in the PN lesion [16], implying that the expression pattern and molecular mechanism of SERPINB3/4 in PN have a common basis with AD and psoriasis. Interestingly, only Th22 cells but not Th17 immune polarization was observed in the circulating blood of PN patients [16]. Increased SERPINB4 expression in psoriatic lesions and serums was positively correlated with disease severity, and serum SERPINB4 was closely correlated with serum IL22, but not IL‐17A or IL‐36γ [15]. The peripheral immune pattern of PN is similar to that in psoriasis, but distinguished from Th2‐dominated AD, partly accounting for the increased expression of SERPINB3/4 in PN patients with increased nodules not patients with severe pruritus. Previous studies have demonstrated that SERPINB3/4 overexpression in HaCaT cells leads to the upregulation of multiple pro‐inflammatory chemokines and cytokines, including *CCL2*, *CCL5*, *CCL20*, *CXCL1*, *CXCL2*, *CXCL5*, *CXCL8*, *CXCL10*, *CXCL11*, *IL1A*, *IL1B*, *IL6*, and *TNF‐α*. Many of these molecules, such as *CCL2*, *CCL5*, *CXCL2*, *CXCL10*, *CXCL11*, *IL1B*, *IL6*, and *TNF‐α*, were known to be regulated by NF‐κB signaling [28]. Belzberg et al and Tsoi et al reported elevated expression of *CCL20*, *CXCL1*, *CXCL5*, *CXCL8*, *CXCL10*, *CXCL11*, *IL1A*, and *IL1B* in PN lesions [16, 29]. At the protein level, Belzberg et al. confirmed the upregulation of IL‐1A in PN epidermis [16]. While we did not directly measure NF‐κB targets in our PN lesions, the overlapping upregulation of NF‐κB‐dependent chemokines and cytokines (e.g., *CXCL10*, *CXCL11*, *IL1B*) in PN lesions suggests a similar inflammatory process may be at play. This parallel further supports the potential role of SERPINB3/4 in driving inflammation in PN.

The correlation between SERPINB3/4 and PN severity was observed primarily with IGA and not with PAS. We speculate that IGA captures the extent and intensity of skin lesions, NRS focuses solely on pruritus intensity. SERPINB3/4 may be more closely associated with the structural changes following scratch (e.g., epidermal dysfunction and fibrosis) rather than the sensory component of itch, which aligns with its known involvement in epidermal dysfunction and immune regulation. Future studies should explore the relationship between SERPINB3/4 and other clinical parameters, such as itch mediators (e.g., IL‐31, TSLP) and neuronal activation markers, to better understand its role in pruritus.

Assessing the disease activity and severity in PN is vital in preventing nodule formation and providing effective treatment. In addition to the commonly used inflammation indicators CRP and ESR, some emerging inflammatory indicators based on immune cells have a good correlation in the assessment of the disease activity in inflammatory diseases and response to treatment, such as ELR, NLR, SII and PIV [30, 31, 32, 33, 34]. PN and AD both belong to type 2 inflammatory skin diseases, indicating a similar expression pattern of type 2 biomarkers may be present in these two disorders. Previous evidence has shown that eosinophil count and total IgE levels were significantly increased in the peripheral blood of AD patients compared with HCs [26]. Peripheral eosinophil levels and ELR were parallel with the disease severity of AD [35, 36]. Our results showed that ELR and the count and percentage of peripheral eosinophils were significantly enhanced in PN patients with IGA 4 compared with the group IGA 3, indicating that eosinophils‐related indexes may be parallel with the disease severity of PN. Basophils act upstream in the type‐2‐cell‐mediated immune response cascade [37]. We found that the percentage of basophils and BLR were elevated in the peripheral blood from PN patients with IGA 4 compared with the group IGA 3, possibly in preparation for initiating a type 2 inflammatory response in the lesion. The activation of basophils may be in an IgE‐independent fashion since no significance was shown in total IgE levels between the group IGA 3 and IGA 4.

While our study provides novel insights into the expression of SERPINB3/4 in moderate‐to‐severe PN and its correlation with type 2 inflammatory markers, several limitations should be acknowledged. First, although we identified correlations between SERPINB3/4 and type 2 inflammatory markers, The underlying molecular mechanisms remain poorly understood. Second, the sample size in our study was relatively small, which may restrict the generalizability of our findings and reduce the statistical power to identify differences in SERPINB3/4 expression among subgroups. Additionally, the absence of matched skin biopsy samples from patients with the highest serum SERPINB3/4 levels may have limited a thorough analysis of tissue‐specific expression patterns. Finally, the use of promising inflammatory indicators such as ELR, NLR, SII, and PIV, requires further standardization and validation in larger, diverse populations to confirm their reliability and clinical applicability in PN and other inflammatory skin diseases.

Future exploration is needed to understand the role of SERPINB3/4 in keratinocyte dysfunction, dermal fibrosis, and inflammatory cell infiltration in PN. It is necessary to clarify the regulatory pathways involving Th2, Th17, and Th22 cytokines in PN circulation and lesions and their impact on SERPINB3/4 expression. Comparative analyses between PN, AD and psoriasis could provide insights into the shared and distinct molecular mechanisms of SERPINB3/4 across these conditions. Large‐scale, multicenter studies are essential to validate SERPINB3/4 as a reliable biomarker for assessing PN disease activity and severity. Finally, identifying therapeutic targets within the SERPINB3/4 pathway holds promise for developing novel and effective treatments for PN and related inflammatory skin disorders.

## 5 Conclusion

This first study on SERPINB3/4 expression in moderate‐to‐severe PN patients revealed a notable elevation in SERPINB3/4 levels in both serum and lesions. Serum SERPINB3/4 levels were positively correlated with IGA scores and peripheral eosinophil‐related parameters, suggesting a potential role for SERPINB3/4 in PN pathogenesis. However, further validation in larger cohorts using additional severity measures, such as PAS and quality of life scales, is needed to confirm its utility as a biomarker for PN.

## Author Contributions

**Jiali Wu:** conceptualization, data curation, writing – original draft. **Kaoyuan Zhang:** investigation. **Yan Liao:** resources. **Chener Yang:** resources. **Jingyao Ge:** visualization. **Chenchen Wu:** visualization. **Bo Yu:** funding acquisition. **Weilong Zhong:** funding acquisition. **Yong Shao:** writing – review and editing, supervision. **Xia Dou:** funding acquisition, writing – review and editing, supervision.

## Ethics Statement

The patients/participants provided their written informed consent to participate in this study. The study was approved by the Ethical Committee of Peking University Shenzhen Hospital.

## Conflicts of Interest

The authors declare no conflicts of interest.

## Supporting information


**Figure S1:** SERPINB3/4 expression was significantly increased in PN lesions. (a) IHC of SERPINB3/4 on skin sections from HC skin (*n* = 3) and PN lesions (*n* = 3). (b) The percentage of SERPINB3/4‐positive area was calculated using ImageJ software and compared between healthy skin and PN lesions. Scale bar = 100 μm, original magnification ×20. Data are presented as means ± SEM. ***p < 0.001. HC, healthy control; PN, prurigo nodularis; IHC, immunohistochemistry.

**Figure S2:** Serum SERPINB3/4 expression in PN patients with and without an atopic history. Serum SERPINB3/4 expression performed by ELISA in PN patients with an atopic history (*n* = 20) and without an atopic history (*n* = 19). Data are presented as means ± SEM. Ns: no significance. PN, prurigo nodularis.

## Data Availability

The data that support the findings of this study are available from the corresponding author upon reasonable request.
