# Supplementary figures and images for: Serum SERPINB3/4 in Moderate‐to‐Severe Prurigo Nodularis: A Potential Biomarker for Disease Severity and Eosinophil‐Related Parameters
Source: Immun Inflamm Dis. 2025 Sep 25;13(9):e70263. doi: 10.1002/iid3.70263 (PMC12461664; doi:10.1002/iid3.70263)

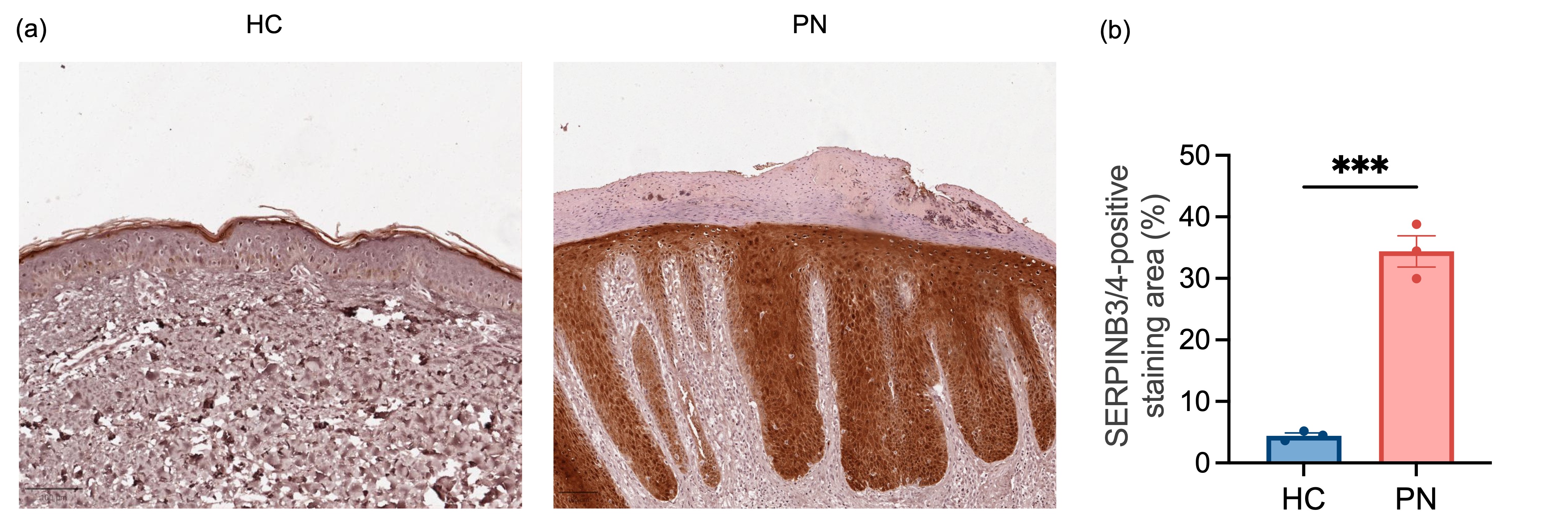

Supplement: Supplementary file 1 — Figure S1: SERPINB3/4 expression was significantly increased in PN lesions. (a) IHC of SERPINB3/4 on skin sections from HC skin (n = 3) and PN lesions (n = 3). (b) The percentage of SERPINB3/4‐positive area was calculated using ImageJ software and compared between healthy skin and PN lesions. Scale bar = 100 μm, original magnification ×20. Data are presented as means ± SEM. ***p < 0.001. HC, healthy control; PN, prurigo nodularis; IHC, immunohistochemistry. [file IID3-13-e70263-s001.jpg]

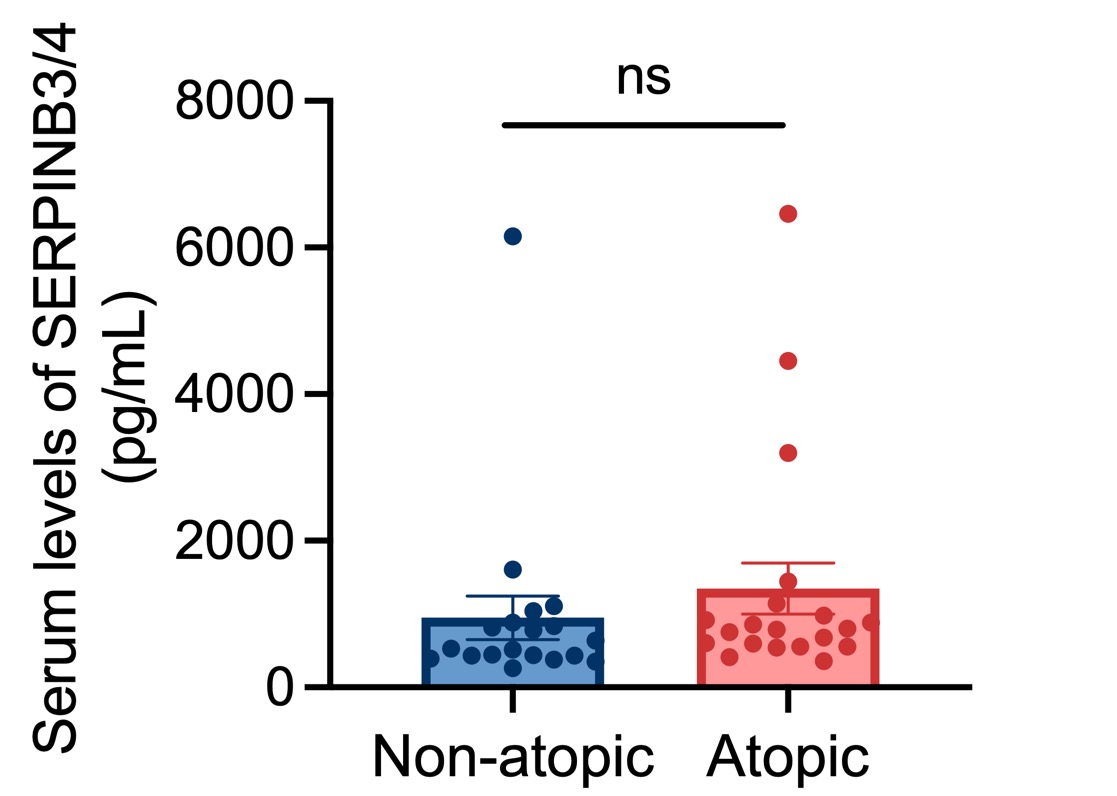

Supplement: Supplementary file 2 — Figure S2: Serum SERPINB3/4 expression in PN patients with and without an atopic history. Serum SERPINB3/4 expression performed by ELISA in PN patients with an atopic history (n = 20) and without an atopic history (n = 19). Data are presented as means ± SEM. Ns: no significance. PN, prurigo nodularis. [file IID3-13-e70263-s002.jpg]
